# “Hypochondriac” discourse in the modern society: a way to self-care about health or health anxiety?

**DOI:** 10.1192/j.eurpsy.2022.467

**Published:** 2022-09-01

**Authors:** A. Tkhostov, E. Rasskazova, V. Emelin

**Affiliations:** 1 Moscow State University, Clinical Psychology, Mokhovaja, Russian Federation; 2 Mental Health Research Center, Medical Psychology, Moscow, Russian Federation; 3 Moscow State University, Clinical Psychology, Moscow, Russian Federation

**Keywords:** “Hypochondriac”, discourse, self-care, health behavior

## Abstract

**Introduction:**

Modern social discourse emphasizes an importance of health either as a limiting resource that needs to be “saved” and “restored”, or as a vulnerability that should be protected, or as a “natural gift” that needs support and recovery by natural methods including alternative medicine. Advertisement adds to the social discourse a meaning of health as a sign of success. Research demonstrated that beliefs in any of these meanings is associated with higher adherence to medical recommendations but also higher catastrophizing of bodily sensations, somatosensory amplification and belief in bodily weakness (Rasskazova et al., 2017).

**Objectives:**

To reveal relationships of beliefs and thoughts in “hypochondriac discourse” with subjective importance of health self-care and health-oriented behavior.

**Methods:**

340 participants 17-77 years old filled “Hypochondriac” Discourse Questionnaire (Rasskazova et al., 2016) that includes four scales measuring beliefs and four scales measuring frequency of thoughts about each health meaning, and Health Self-Care Scale (Rasskazova et al., 2021) that differentiates subjective importance of different ways of self-care and activities (Cronbach’s alphas .66-.80).

**Results:**

All beliefs in “hypochondriac discourse” except importance of alternative medicine are related to medical health monitoring and active styles of life (r=.23-.43) but unrelated to reported activities. Frequency of thoughts about “hypochondriac discourse” are related to adherence to health behavior (r=.31-.49).

**Conclusions:**

Frequent thoughts about “hypochondriac discourse” could be protective factor helping to support active life styles but also could lead to over-protection in healthy people. Research is supported by the Russian Foundation for Basic Research, project No. 22-28-01643

**Disclosure:**

Research is supported by the Russian Foundation for Basic Research, project No. 22-28-01643

